# Transient Right Temporal Visual Field Loss Temporally Associated with Maintenance Infliximab in Crohn’s Disease: A Case Report with Focused Literature Review

**DOI:** 10.1159/crg/abwag002

**Published:** 2026-08-11

**Authors:** Asad Shahbaz, Suliman AlMohtasib, Fatima Sajjadi, Fadel Alqatati, Nagham Dhiaa Sadik

**Affiliations:** Department of Internal Medicine, Hamad Medical Corporation, Doha, Qatar

**Keywords:** Crohn’s disease, Infliximab, Visual field loss, Optic neuritis, Neuro-ophthalmic adverse event, Case report

## Abstract

**Introduction:**

Infliximab is an established therapy for Crohn’s disease, but uncommon neuro-ophthalmic adverse events have been reported. Most published infliximab-associated visual complications have involved optic neuritis or optic neuropathy rather than transient, structurally unexplained visual field symptoms. We report a 32-year-old man with ileocolonic Crohn’s disease in remission on maintenance infliximab who developed sudden right temporal visual field loss approximately 2 days after infusion.

**Case Presentation:**

The episode of sudden right temporal visual field loss after infliximab infusion lasted 15–30 min and was followed by blurred vision in the same field for about 6 h, with mild periorbital pain and headache. Ophthalmologic examination, including funduscopy, was unremarkable. Computed tomography of the head and magnetic resonance imaging of the brain and orbits with contrast were also unremarkable. Electrocardiography showed normal sinus rhythm, and transthoracic echocardiography demonstrated normal biventricular function without evidence of patent foramen ovale. Symptoms resolved spontaneously. The patient also described a prior episode of transient right-sided limb weakness occurring after a previous infliximab exposure, suggesting a recurrent transient neurological pattern.

**Conclusion:**

Although causality cannot be established from a single case, this presentation appears to represent a recurrent, transient, imaging-negative neuro-ophthalmic syndrome temporally associated with maintenance infliximab exposure. The case underscores the need to exclude vascular, inflammatory, demyelinating, and migrainous causes while remaining alert to uncommon transient neurological events in patients receiving anti-tumor necrosis factor therapy.

## Introduction

Ocular and neuro-ophthalmic complications in inflammatory bowel disease are uncommon but clinically important because they may reflect either extraintestinal disease, treatment-related toxicity, or unrelated vascular and inflammatory pathology [1]. Optic nerve involvement in inflammatory bowel disease may occur as optic neuritis, ischemic optic neuropathy, or other inflammatory orbital syndromes, and anti-tumor necrosis factor therapy has also been implicated in demyelinating and optic nerve adverse events [1, 2]. Infliximab-associated visual complications reported to date have most often involved optic neuritis or optic neuropathy with structural or clinical evidence of optic nerve involvement [2–8]. A recent report described transient visual loss during the first infliximab infusion in Crohn’s disease, suggesting that not all events follow a classic inflammatory or demyelinating pattern [9].

Beyond optic nerve involvement, anti-tumor necrosis factor therapy has been associated with a broader spectrum of rare but clinically significant neurological adverse events. These include central nervous system demyelination presenting as a multiple sclerosis-like illness, peripheral neuropathies such as Guillain-Barré syndrome and chronic inflammatory demyelinating polyneuropathy, and longitudinal extensive transverse myelitis (LETM) – a severe spinal cord syndrome characterized by MRI lesions extending over three or more vertebral segments [10, 11]. Published case reports and review articles have documented LETM occurring in patients receiving infliximab and other anti-TNF agents, often necessitating drug discontinuation and aggressive immunosuppressive treatment [10, 11]. The pathophysiological basis of these adverse events remains incompletely understood but may involve paradoxical upregulation of pro-inflammatory mediators, disruption of the blood-brain and blood-retinal barriers, and promotion of demyelinating processes through TNF-mediated modulation of myelin-reactive lymphocyte populations [12]. Notably, anti-TNF agents including infliximab and adalimumab are also employed therapeutically for refractory orbital inflammatory conditions such as orbital myositis [13], which underscores the context-dependent nature of the risk-benefit balance associated with TNF inhibition in the orbital and neurological compartments.

We describe a patient with Crohn’s disease in remission who developed transient right temporal visual field loss shortly after maintenance infliximab exposure, with unrevealing ophthalmologic and neuroimaging investigations, spontaneous recovery, and a history of a prior transient focal neurological episode after an earlier infusion. This case is best framed as a case report with focused literature review describing a recurrent, transient, imaging-negative neuro-ophthalmic syndrome temporally associated with maintenance infliximab exposure.

## Case Presentation

A 32-year-old Syrian man with ileocolonic Crohn’s disease complicated by perianal fistulae and abscesses presented with sudden loss of vision involving the right temporal visual field. He described the deficit as complete blackness in the affected field, lasting approximately 15–30 min, followed by blurred vision in the same location for about 6 h. The episode was accompanied by mild right periorbital pain and frontal headache. He denied fever, chills, nausea, neck pain, loss of consciousness, seizure, trauma, diplopia, facial deviation, sensory symptoms, ataxia, chest pain, palpitations, dyspnea, abdominal pain, diarrhea, and urinary symptoms. He had received his last infliximab infusion 2 days earlier and was on maintenance infliximab 400 mg intravenously every 8 weeks.

The patient described a previous transient neurological event several months earlier, again beginning approximately 2 days after an infliximab infusion, during which he experienced sudden right-sided limb weakness lasting about 24 h and resolving spontaneously without medical assessment. This history of a prior focal neurological episode occurring in the same temporal relationship to infliximab exposure was considered clinically relevant to the current presentation.

His Crohn’s disease had been diagnosed more than a decade earlier. Initial therapy consisted of 5-aminosalicylic acid and azathioprine, after which treatment was escalated to infliximab because of bone marrow suppression attributed to azathioprine. He later achieved prolonged remission, and infliximab was discontinued. Several years later, he developed recurrent perianal symptoms, and subsequent colonoscopy demonstrated ulceration of the ileocecal valve and terminal ileum. Histopathology confirmed moderate chronic active inflammation with ulceration, cryptitis, and crypt distortion in the terminal ileum, without granulomas, dysplasia, or malignancy. Infliximab was therefore reintroduced at a standard induction regimen (5 mg/kg at weeks 0, 2, and 6) followed by maintenance dosing at 400 mg intravenously every 8 weeks. At the time of the visual event, the patient had received over 25 infusions during the current maintenance course. At his most recent gastroenterology follow-up before this event, the disease was in remission on infliximab, with normal fecal calprotectin and a therapeutic infliximab level during maintenance therapy.

On examination, he was hemodynamically stable and in no acute distress. Ophthalmologic assessment showed best-corrected visual acuity of 6/6 in both eyes and intraocular pressure of 14 mm Hg bilaterally. Anterior segment examination was unremarkable, and funduscopy demonstrated normal optic discs and maculae with flat retinas bilaterally. The ophthalmology team concluded that the ocular findings did not explain the visual symptoms. Neurological examination was normal, with intact cranial nerves, full power in all limbs, preserved sensation, normal coordination, and normal gait. Routine laboratory investigations were unremarkable apart from a mildly elevated hematocrit of 50.5% and red blood cell count of 5.8 × 10^6^/μL.

Computed tomography of the head showed no acute intracranial abnormality. Magnetic resonance imaging of the brain and orbits with contrast showed no focal diffusion restriction, intracranial hemorrhage, mass lesion, or pathological post-contrast enhancement. The cavernous sinuses were grossly unremarkable, and no abnormal signal or enhancement was identified along either optic nerve. Cardiac evaluation was also unrevealing: electrocardiography showed normal sinus rhythm, and transthoracic echocardiography demonstrated normal biventricular size and function, a left ventricular ejection fraction of 55%, no regional wall motion abnormality, normal pulmonary artery pressure, and no evidence of a patent foramen ovale.

Given the acute presentation, the differential diagnosis included toxic anterior optic neuropathy, optic neuritis, and central neurological causes such as demyelinating disease (including multiple sclerosis) and vascular events (including transient ischemic attack and posterior circulation ischemia). The patient was admitted for observation and further evaluation. After normal ophthalmologic examination and unrevealing neuroimaging, the working neurological impression was transient visual loss possibly related to infliximab, with migraine with aura retained as an alternative diagnosis. Gastroenterology also reviewed the case because of the temporal relationship between symptom onset and recent infliximab exposure. No immediate inpatient modification of Crohn’s disease therapy was made, and infliximab remained on the discharge medication list.

His visual symptoms resolved spontaneously, and he returned to baseline with intact visual fields. He was discharged home in stable condition. At subsequent outpatient follow-up, biologic therapy was continued, and he received additional two infusions without documented recurrence of visual or focal neurological symptoms. This case report has been reported in line with the CARE guidelines.

## Discussion

The central clinical challenge this case illustrates is how to interpret and manage transient, structurally unexplained neuro-ophthalmic events occurring in temporal association with maintenance anti-TNF therapy. The absence of ophthalmologic, neuroimaging, and cardiac structural abnormalities prevents attribution to a defined anatomical lesion, leaving the underlying mechanism speculative. Nonetheless, the recurrence of a distinct transient focal neurological episode after a separate infliximab infusion, with a consistent 2-day latency, suggests that these events may not be entirely coincidental. The clinical implication is that such presentations require systematic exclusion of vascular, inflammatory, and demyelinating etiologies, followed by individualized reassessment of ongoing biologic therapy rather than automatic discontinuation. The fact that infliximab was continued after discharge and the patient remained asymptomatic at follow-up further illustrates the complexity of causal attribution in this setting and supports the possibility that not all anti-TNF-associated transient neurological events necessitate permanent drug discontinuation.

The differential diagnosis of transient visual field loss in this context can be organized across three domains. First, general causes of transient monocular or hemianopic visual loss include retinal or optic nerve ischemia, embolic amaurosis fugax, vasospasm, migraine-related phenomena, demyelinating disease, and other transient neuro-ophthalmic syndromes; such presentations are approached as vascular emergencies until structural causes are excluded [14]. Second, ocular manifestations of inflammatory bowel disease itself, independent of anti-TNF therapy, may include episcleritis, uveitis, and, less commonly, optic neuritis or ischemic optic neuropathy, reflecting the systemic inflammatory burden of the underlying disease [1, 7, 8]. Third, anti-TNF-associated ocular and neurological complications represent a distinct category, encompassing optic neuritis, optic neuropathy, demyelinating syndromes, and rarer events such as LETM [2–8, 10, 11]. Distinguishing anti-TNF-associated retinal or ocular migraine from a direct drug effect is particularly challenging. Retinal migraine, as defined by International Headache Society criteria, requires fully reversible monocular visual symptoms associated with headache and no better alternative explanation, typically without a structural correlate [15]. The transient temporal field deficit accompanied by headache in the present case is consistent with a migrainous mechanism; however, the recurrence of a separate focal neurological episode following a prior infliximab infusion makes an isolated primary headache disorder a less satisfying explanation, and anti-TNF-associated immune-mediated or vasospastic mechanisms remain part of the differential.

A transient vasospastic or ischemic mechanism remains possible, particularly given the sudden onset and negative symptom profile, although no objective vascular lesion was identified during admission. The normal electrocardiogram and echocardiogram without patent foramen ovale make a cardioembolic mechanism less likely, but they do not exclude all vascular etiologies. Against classic optic neuritis, however, this patient had normal fundus findings, unrevealing brain-orbit magnetic resonance imaging, rapid spontaneous recovery, and no documented persistent visual deficit. A delayed infusion-related or immune-mediated phenomenon is conceivable, particularly because symptoms occurred 2 days after infusion rather than during infusion itself. Accordingly, the most defensible interpretation is not proven infliximab neurotoxicity, but a recurrent transient syndrome in temporal association with infliximab exposure.

The temporal relationship between anti-TNF administration and onset of optic or neurological complications is variable across published reports. Optic neuritis has been documented as early as the third infusion [3] and as late as several months into maintenance therapy [6], suggesting heterogeneous immune-mediated mechanisms rather than a single pharmacological pathway. Events occurring within days of infusion – as in the present case – may reflect a delayed immunological perturbation, transient disruption of the blood-retinal or blood-brain barrier, or vasospastic phenomena distinct from classic inflammatory demyelination. Under physiological conditions, TNF-α contributes to barrier integrity and suppression of autoreactive lymphocyte populations; its systemic blockade may paradoxically promote neuroinflammatory processes in susceptible individuals through upregulation of myelin-reactive T cells and altered cytokine homeostasis [12]. Regarding rechallenge with infliximab following ophthalmologic complications, the published evidence is limited. Most reported cases involving confirmed optic neuritis have led to permanent drug discontinuation without reintroduction [2–8]. In cases of transient, imaging-negative events without confirmed structural neuritis, however, continued or resumed therapy with close monitoring has been described as a potential strategy [9]. The present case is instructive in this regard: infliximab was continued after discharge, and the patient remained free of recurrent neurological symptoms at follow-up, supporting the view that management should be individualized according to event severity, reversibility, and the importance of ongoing biologic therapy for disease control.

The focused literature review helps position this case. Anti-tumor necrosis factor-associated optic neuritis and optic neuropathy have been reported for more than 2 decades [2–8]. The spectrum of previously published visual adverse events and their comparison with the present case are summarized in Table 1. Early reports described bilateral anterior toxic optic neuropathy after infliximab, including onset after a third dose [3]. Retrobulbar optic neuritis has been reported in Crohn’s disease approximately 4 weeks after the third infusion, with improvement after corticosteroid therapy and drug withdrawal [4]. Chan and Castellanos described possible bilateral anterior optic neuropathy during a third infliximab infusion in Crohn’s esophagitis [5]. Aasly et al. [6] reported bilateral optic neuritis after several months of infliximab therapy, with recovery after corticosteroid treatment. More severe and even irreversible optic neuritis has also been reported [7, 8]. In inflammatory bowel disease specifically, Alexandre et al. [7] emphasized that optic neuritis may occur both with and without anti-tumor necrosis factor exposure, which complicates attribution in individual cases. Hsieh et al. [8] further showed that inflammatory bowel disease itself is associated with a higher risk of optic neuritis than the general population, again underscoring the difficulty of separating disease-related from treatment-related mechanisms.

**Table 1.** Review of literature: neurological and ocular adverse events associated with infliximab

Author (Year)	Phenotype	Indication	Timing	Exposure	Imaging findings	Recurrence	Management	Outcome	Difference from present case
ten Tusscher et al. [3] (2003)	Bilateral anterior toxic optic neuropathy	RA	After 3rd dose	Early	Anterior optic neuropathy	Not emphasized	Incompletely reported	Incompletely reported	Present case: transient field loss, normal imaging
Ouakaa-Kchaou et al. [4] (2009)	Retrobulbar optic neuritis	CD	4 weeks after 3rd dose	Early	Structural inflammatory syndrome	No	Steroids; IFX cessation	Favorable	Present case: 2 days post-infusion; no structural neuritis
Chan &and Castellanos [5] (2010)	Possible bilateral anterior optic neuropathy	CD Esophagitis	During 3rd infusion	Early	Bilateral anterior optic neuropathy	No	Details limited	Recovered	Present case: maintenance exposure; spontaneous resolution
Aasly [6] (2011)	Bilateral optic neuritis	PsA	∼6 months	Maintenance	Optic neuritis; normal MRI	No	Corticosteroids	Recovery (10 weeks)	Present case: unilateral; more transient
Alexandre et al. [7] (2016)	Optic neuritis in IBD	IBD	Variable	Mixed	Heterogeneous	Variable	Heterogeneous	Heterogeneous	Present case: imaging-negative; temporally linked to maintenance
Dermawan et al. [8] (2020)	Mono-ocular visual field loss	UC	During treatment	Ongoing	Optic neuritis	No	ON-directed Rx	Not reported	Present case: lacked objective neuritis on funduscopy/MRI.
Coelho et al. [9] (2025)	Transient visual loss	CD	During 1st infusion	Induction	Transient event; no structural findings	No	Infusion management	Recovered	Present case: maintenance exposure; prior focal neuro event
Present case	Transient right temporal field loss; blurring	CD	2 days post-infusion	Maintenance	Normal ophthalmology, CT, MRI, ECG, TTE	Possible (prior weakness)	Observation; IFX continued	Spontaneous recovery	Index case

CD, Crohns disease; ECG, electrocardiogram; IBD, inflammatory bowel disease; IFX, infliximab; ON, optic neuritis; PsA, psoriatic arthritis; RA, rheumatoid arthritis; TTE, transthoracic echocardiography; UC, ulcerative colitis.

The most directly comparable publication is the recent case by Coelho et al. [9] which described transient visual loss during the first infliximab infusion in Crohn’s disease. The present case differs in several potentially meaningful ways. The event occurred during maintenance therapy rather than first exposure, the dominant phenotype was a transient right temporal visual field deficit rather than generalized asymmetric visual impairment, structural investigations remained unrevealing, electrocardiography and echocardiography were normal, and the patient described a previous separate transient focal neurological event after another infusion. In addition, infliximab was continued after discharge and the patient remained asymptomatic at follow-up, which further emphasizes the complexity of attribution in transient neuro-ophthalmic events associated with biologic therapy. Thus, the manuscript’s contribution lies less in claiming a wholly new adverse effect and more in describing a distinct phenotype and recurrence pattern that broadens clinical awareness of possible transient neuro-ophthalmic events temporally associated with infliximab.

The limitations are substantial and should be stated clearly. Monocularity was based on symptom description rather than formal perimetry. No post-event optical coherence tomography, color vision testing, relative afferent pupillary defect documentation, retinal angiography, or carotid imaging was available in the records reviewed. The prior episode of limb weakness was patient-reported and was not medically assessed at the time it occurred. Although infliximab was continued and no recurrent symptoms were documented at follow-up, the patient was lost to follow-up due to travel back to home country. These limitations prevent any firm conclusion regarding mechanism or causality.

The practical learning point is that sudden transient visual symptoms after infliximab should not automatically be dismissed as benign, but neither should they be framed prematurely as definitive infliximab neurotoxicity. Importantly, transient neurological or visual symptoms temporally associated with anti-TNF therapy may not necessarily require permanent discontinuation of the agent. In the present case, the patient experienced complete visual recovery without specific treatment and tolerated continued infliximab exposure before and after separate episodes without progression to severe adverse effects. Clinicians should urgently exclude structural, vascular, inflammatory, and demyelinating causes and then reassess the risk-benefit profile of ongoing anti-tumor necrosis factor therapy in a multidisciplinary setting, with particular attention to the transient or progressive nature of the neurological event and the necessity of ongoing biologic therapy for disease control.

## Conclusion

This case appears to represent a recurrent, transient, imaging-negative neuro-ophthalmic syndrome temporally associated with maintenance infliximab exposure in a patient with Crohn’s disease. The recurrence pattern, unrevealing structural investigations, and benign course after continuation of therapy support careful clinical recognition, urgent exclusion of alternative causes, and individualized multidisciplinary reassessment of ongoing treatment.

## Statement of Ethics

Ethical approval is not required for this case report in accordance with local or national guidelines. Written informed consent was obtained from the patient for publication of the details of their medical case. A completed CARE checklist is provided as online supplementary material (for all online suppl. material, see https://doi.org/10.1159/crg/abwag002).

## Conflict of Interest Statement

The authors have no conflicts of interest to declare.

## Funding Source

Open access funding was provided by the Qatar National Library for publishing of this case report.

## Author Contributions

Asad Shahbaz conceived and coordinated the case report, drafted the manuscript, and was responsible for the overall writing, editing, and submission of the manuscript. Fatima Sajjadi contributed to writing the Discussion section. Suliman AlMohtasib contributed to writing the Case Presentation section. Fadel Alqatati conducted the literature review. Nagham Dhiaa Sadik provided supervision and oversight of the manuscript. All authors reviewed and approved the final version of the manuscript.

## Data Availability

All data generated or analyzed during this study are included in this article and its supplementary material files. Further inquiries can be directed to the corresponding author.

## References

[1] Katsanos A , Asproudis I, Katsanos KH, Dastiridou AI, Aspiotis M, Tsianos EV. Orbital and optic nerve complications of inflammatory bowel disease. J Crohns Colitis. 2013;7(9):683–93. 10.1016/j.crohns.2012.09.020 23083697

[2] Simsek I , Erdem H, Pay S, Sobaci G, Dinc A. Optic neuritis occurring with anti-tumour necrosis factor alpha therapy. Ann Rheum Dis. 2007;66(9):1255–8. 10.1136/ard.2006.066787 PMC1955135 17456525

[3] ten Tusscher MPM , Jacobs PJC, Busch MJWM, de Graaf L, Diemont WL. Bilateral anterior toxic optic neuropathy and the use of infliximab. BMJ. 2003;326(7389):579. 10.1136/bmj.326.7389.579 PMC151521 12637403

[4] Ouakaa-Kchaou A , Gargouri D, Trojet S, Hefaiedh R, Elloumi H, Kochlef A, . Retrobulbar optic neuritis associated with infliximab in a patient with Crohn’s disease. J Crohns Colitis. 2009;3(2):131–3. 10.1016/j.crohns.2009.02.007 21172257

[5] Chan JW , Castellanos A. Infliximab and anterior optic neuropathy: case report and review of the literature. Graefes Arch Clin Exp Ophthalmol. 2010;248(2):283–7. 10.1007/s00417-009-1227-y 19916016

[6] Aasly JO . Bilateral optic neuritis associated with the use of infliximab. Case Rep Ophthalmol Med. 2011;2011:232986. 10.1155/2011/232986 PMC3350263 22611506

[7] Alexandre B , Vandermeeren Y, Dewit O, Moreels T, de Boer N, Dhar A, . Optic neuritis associated or not with tumour necrosis factor antagonists in patients with inflammatory bowel disease. J Crohns Colitis. 2016;10(5):541–8. 10.1093/ecco-jcc/jjw003 PMC4957451 26759360

[8] Hsieh YH , Chung CH, Sun CA, Chen PH, Chen YH, Liang CM, . Association between optic neuritis and inflammatory bowel disease: a population-based study. J Clin Med. 2021;10(4):688. 10.3390/jcm10040688 PMC7916645 33578895

[9] Coelho H , Dias MT, Oliveira AM. Transient visual loss due to infliximab administration: a new adverse event to biologic therapy in Crohn’s disease. ACG Case Rep J. 2025;12(12):e01940. 10.14309/crj.0000000000001940 PMC12721776 41438717

[10] Mohan N , Edwards ET, Cupps TR, Oliverio PJ, Sandberg G, Crayton H, . Demyelination occurring during anti-tumor necrosis factor alpha therapy for inflammatory arthritides. Arthritis Rheum. 2001;44(12):2862–9. 10.1002/1529-0131(200112)44:12<2862::aid-art474>3.0.co;2-w 11762947

[11] Stojsavljevic J , Perez RR, Petcu E, Odenigbo C, Madrid C, Dumic I, . Steroid refractory and plasma exchange partially responsive longitudinally extensive transverse myelitis due to tumor necrosis factor-alpha (etanercept): a case report. Clin Pract. 2026;16(5):86. 10.3390/clinpract16050086 PMC13206227 42187520

[12] Kemanetzoglou E , Andreadou E. CNS demyelination with TNF-α blockers. Curr Neurol Neurosci Rep. 2017;17(4):36. 10.1007/s11910-017-0742-1 PMC5364240 28337644

[13] Garrity JA , Coleman AW, Matteson EL, Eggenberger ER, Waitzman DM. Treatment of recalcitrant idiopathic orbital inflammation (chronic orbital myositis) with infliximab. Am J Ophthalmol. 2004;138(6):925–30. 10.1016/j.ajo.2004.06.077 15629282

[14] Douglas VP , Rachapudi SS, Davila-Siliezar P, Laylani NAR, Lee AG. Transient monocular visual loss (amaurosis fugax): how does age impact diagnosis? Ophthalmol Ther. 2024;13(6):1417–25. 10.1007/s40123-024-00932-z PMC11109035 38587773

[15] Chong YJ , Mollan SP, Logeswaran A, Sinclair AB, Wakerley BR. Current perspective on retinal migraine. Vis. 2021;5(3):38. 10.3390/vision5030038 PMC8396291 34449754

